# Satellite-based soil moisture provides missing link between summertime precipitation and surface temperature biases in CMIP5 simulations over conterminous United States

**DOI:** 10.1038/s41598-018-38309-5

**Published:** 2019-02-07

**Authors:** A. Al-Yaari, A. Ducharne, F. Cheruy, W. T. Crow, J.-P. Wigneron

**Affiliations:** 1 INRA, UMR, 1391 ISPA Villenave d’Ornon, France; 2 Unité Mixte de Recherche METIS, IPSL, Sorbonne Université, CNRS, EPHE, Paris, France; 3 Laboratoire de Météorologie Dynamique, IPSL, CNRS, Sorbonne Universités, Paris, France; 4 Hydrology and Remote Sensing Lab, USDA ARS, Beltsville, MD USA

## Abstract

Past studies have shown that climate simulations have substantial warm and dry biases during the summer in the conterminous United States (CONUS), particularly in the central Great Plains (CGP). These biases have critical implications for the interpretation of climate change projections, but the complex overlap of multiple land-atmosphere feedback processes make them difficult to explain (and therefore correct). Even though surface soil moisture (SM) is often cited as a key control variable in these processes, there are still knowledge gaps about its specific role. Here, we use recently developed remotely sensed SM products to analyse the link between spatial patterns of summertime SM, precipitation and air temperature biases over CONUS in 20 different CMIP5 simulations. We identify three main types of bias combinations: (i) a dry/warm bias over the CGP region, with a significant inter-model correlation between SM and air temperature biases (*R* = −0.65), (ii) a wet/cold bias in NW CONUS, and (iii) a dry/cold bias in SW CONUS. Combined with irrigation patterns, these results suggest that land-atmosphere feedbacks over the CGP are not only local but have a regional dimension, and demonstrate the added-value of large-scale SM observations for resolving the full feed-back loop between precipitation and temperature.

## Introduction

Climate models rely on coupled simulations of the ocean, land surface, atmosphere, and sea ice systems to better understand the Earth’s climate system and its future change^1^. Important examples are the climate simulations of the Coupled Model Intercomparison Project (CMIP), which have been a major contribution to the Intergovernmental Panel on Climate Change’s (IPCC) assessment reports up to Phase 5 (CMIP5^2^) and the ongoing Phase 6 (CMIP6^3^). Climate models participating in the CMIP project have progressively improved in recent decades^1,4^, but they remain associated with many uncertainties and/or biases, due to residual errors in boundary conditions, inadequate model parameterizations and poorly known processes^2,5–9^. More specifically, pronounced systematic summer warm biases -relative to 2-m near surface air temperature (referred to as air temperature in the following) observations- have been found over many continental areas, including the conterminous United States (CONUS) and, more specifically, the CONUS Central Great Plains (CGP) region^8,10–12^. Since air temperature lies at the centre of critical feedbacks between multiple climate system components, its bias (or error) can influence the overall reliability of the CMIP model simulations and future climate projections^10^. Previous studies attributed these warm land biases to multiple factors including, but not limited to: deficiencies in cloud representation, errors in large-scale atmospheric circulation, misrepresented evaporation, and failure to capture heavy rainfall events^10,13–15^. Nevertheless, these warm biases remain an open research problem that needs to be addressed to improve climate models predictions and projections^3^.

In most studies, surface soil moisture (SM) is considered a central variable as it controls key processes at the land atmosphere interface (e.g., surface heat exchanges and their partitioning, runoff generation, evaporation, and evaporative fraction)^16–18^, which are strongly involved in the generation of the warm bias e.g.^15,19–25^. For instance, according to Boé^26^, SM influences precipitation both directly and indirectly through a modulation of the atmospheric thermodynamic properties. Stéfanon *et al*.^23^ have found that SM had a strong impact on air temperature when analysing the summer heat waves over Western Europe. Likewise, Seneviratne *et al*.^27^ have demonstrated that feedbacks between the atmosphere and SM increase the summer air temperature variability in central and eastern Europe. LeMone *et al*.^28^ have concluded that the latent (LH) and sensible (SH) heat exchanges are highly influenced by SM. Furthermore, Miralles *et al*.^29^ have investigated physical processes underlying the heat waves 2010 in Russia and 2003 in central Europe and found that air temperature was anomalously increased due to progressive SM depletion.

However, to date, in spite of the hypothesized role played by SM at the land-atmosphere interface, the direct examination of SM’s role has been hampered by a lack of large-scale SM observations^30,31^. These limitations have recently been overcome through the availability of global SM products derived from microwave remote sensing^32–34^. These products were first used to identify a preference for afternoon rain over dry soils, not well captured by climate models^35^. However, regarding the link between observed SM and the warm bias produced by climate models, the only studies so far have been limited to a few sites and to a few models e.g.^15^.

Here, we use two recent remotely sensed SM products to evaluate the link between SM, precipitation and air temperature biases found in 20 models (see Supplementary Table S1) participating in CMIP5 over the CONUS during the 1979–2008 period. Note that in this study the objective was not to investigate the causality but rather to examine– for the first time – how remotely sensed SM observations fit into the (existing) understanding of how the air temperature and precipitation biases are linked in the models.

We use the new SM data retrieved from L-band microwave observations from the first-dedicated SM mission SMOS (Soil Moisture and Ocean Salinity) satellite (SMOS-IC product version) over 2010–2016. We also used earlier SM observations (1979–2008) from the first ever long-term satellite-based SM product prepared by the ESA’s Climate Change Initiative (CCI)^36^; http://www.esa-soilmoisture-cci.org/. To make the comparison to the biases in air temperature and precipitation more robust, we also used multiple observational datasets for both variables: (i) Climatic Research Unit (CRU) air temperature and precipitation; (ii) University of Delaware (i.e., “Willmott”) global land air temperature; and (iii) Global Precipitation Climatology Project (GPCP) land precipitation. More details on these datasets and their processing are provided in the Methods section.

## Results

### Spatial link between the summer biases in temperature, precipitation and SM

**Figure 1:**
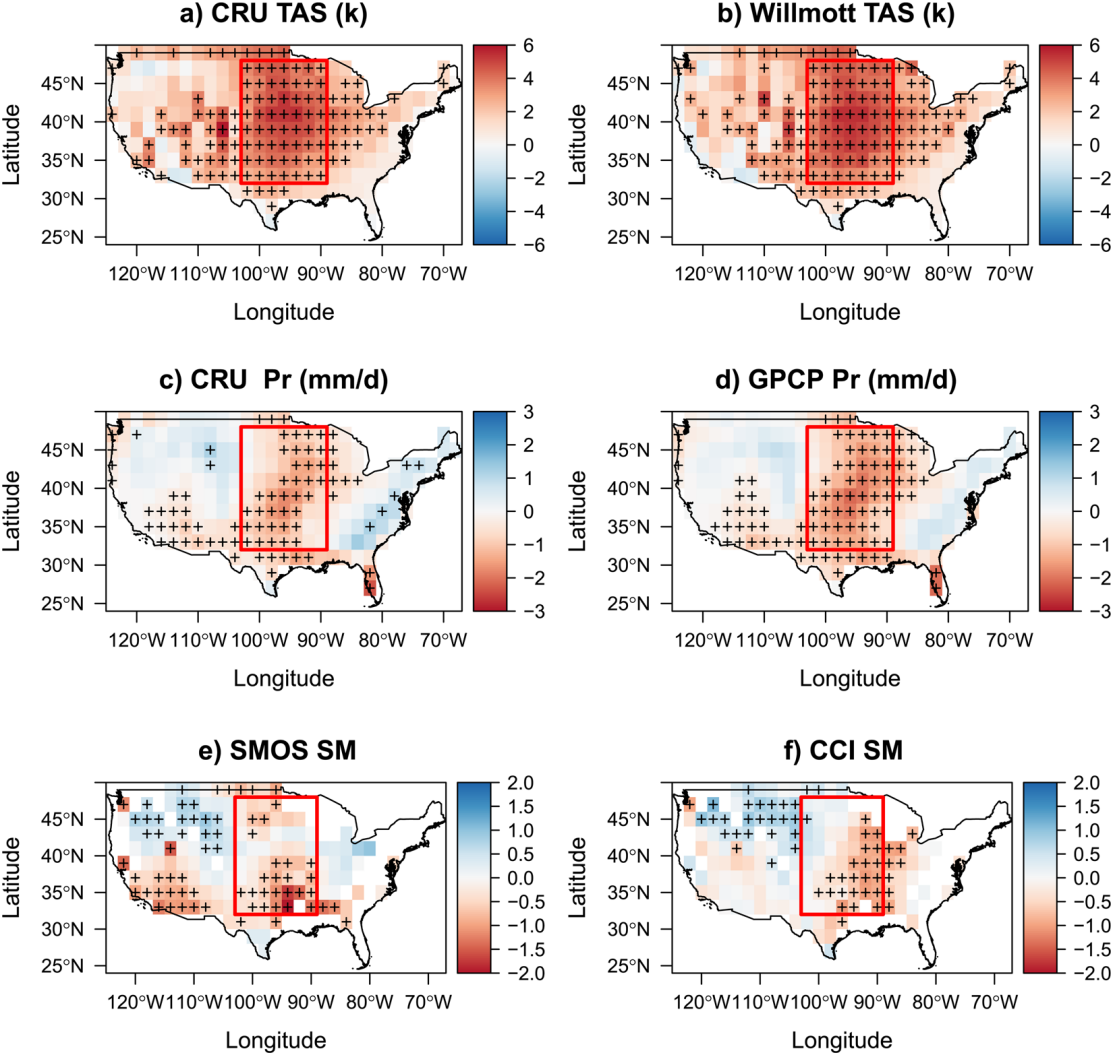
Mean bias between multi-model CMIP5 and observations. (**a**) Multi-model mean bias air temperature (TAS) CMIP5- Obs (CRU). (**b**) Multi-model mean bias TAS CMIP5- Obs (Willmott) during the 1978–2008 period (JJA). (**c**) Multi-model mean bias precipitation (Pr) CMIP5- Obs (CRU) during the 1978–2008 period. (**d**) Multi-model mean bias Pr CMIP5- Obs (GPCP) during the 1978–2008 period (JJA). (**e**) Multi-model mean bias soil moisture (SM) CMIP5- Obs (SMOS; 2010–2016 JJA). (**f**) Multi-model mean bias SM CMIP5- Obs (CCI) during the 1978–2008 period (JJA). Crosses on the figures indicate that at least 65% of the models agree on the sign of the observed bias. White regions represent pixels with percentage of forest >60%, strong topography, or frozen soil conditions, which were excluded from the analyses.

Figure 1a,b shows that the spatial patterns of the mean air temperature bias in CMIP5 simulations are very similar for the CRU and Willmott air temperature observations (see scatter plots of each pair of the different variables in Fig. S1 in the Supplementary). Most of the CMIP5 simulations (more than 2/3; see Supplementary Fig. S2 for each CMIP5 model separately) systematically overestimate both observational datasets, particularly over the CGP region as previously described by Cheruy *et al*.^10^ and Ma *et al*.^37^. However, there are two exceptions (viz. the GISS.E2.R and MRI.AGCM3.2 H models), with low air temperature biases and different spatial patterns compared to the other models (Fig. S2). Note that the singularity of the GISS.E2.R model has already been noted in previous studies e.g.^15^. As for the air temperature biases, there exists a strong similarity in the spatial patterns of the summer mean bias of precipitation for both the CRU and GPCP observational datasets (Figs 1c,d and S3 for each CMIP5 model separately). Compared with the precipitation observations, the CMIP5 models produce excessive precipitation over north-eastern and north-western CONUS and a deficit of precipitation over the CGP region and in south-western CONUS (which is consistent with both Klein *et al*.^38^ and Lin *et al*.^14^).

Reflecting these spatial patterns of precipitation biases, normalized SM is underestimated by a majority of models over the CGP and eastern CONUS in the CMIP5 simulations, while overestimated in north-western CONUS (Fig. 1e,f and Supplementary Figs S4–5 for each CMIP5 model separately). This is consistent with the findings of Yuan & Quiring^39^ who found clear underestimation of SM by the models during summertime over the South Great Plains (SGP) compared to both *in situ* and CCI SM observations. Positive biases are more consistent across models for SM than for the precipitation, particularly over mountainous areas. Some differences can be noted between the SM bias spatial patterns derived from the SMOS and CCI datasets. For instance, over eastern CONUS, the precipitation bias maps are more consistent with the CCI map (although the CCI SM biases are shifted to the south relative to CRU and GPCP precipitation biases). The opposite is true over south-western CONUS (in e.g., Arizona, southern Nevada and southern California) where SMOS-based SM provides a better match to the observed pattern of precipitation bias.

Interestingly, there is a good general agreement (with, again, the exception of GISS.E2.R) between areas of high warm bias and areas of strong precipitation and SM deficits (Figs 1a–f and S2–5 for each model separately). This is particularly notable in central CONUS (including the CGP). However, an area of negative bias in precipitation (for both CRU and GPCP) and SMOS SM in south-western CONUS (with a distinctive triangular shape) is not clearly associated with an overall corresponding warm bias.

**Figure 2:**
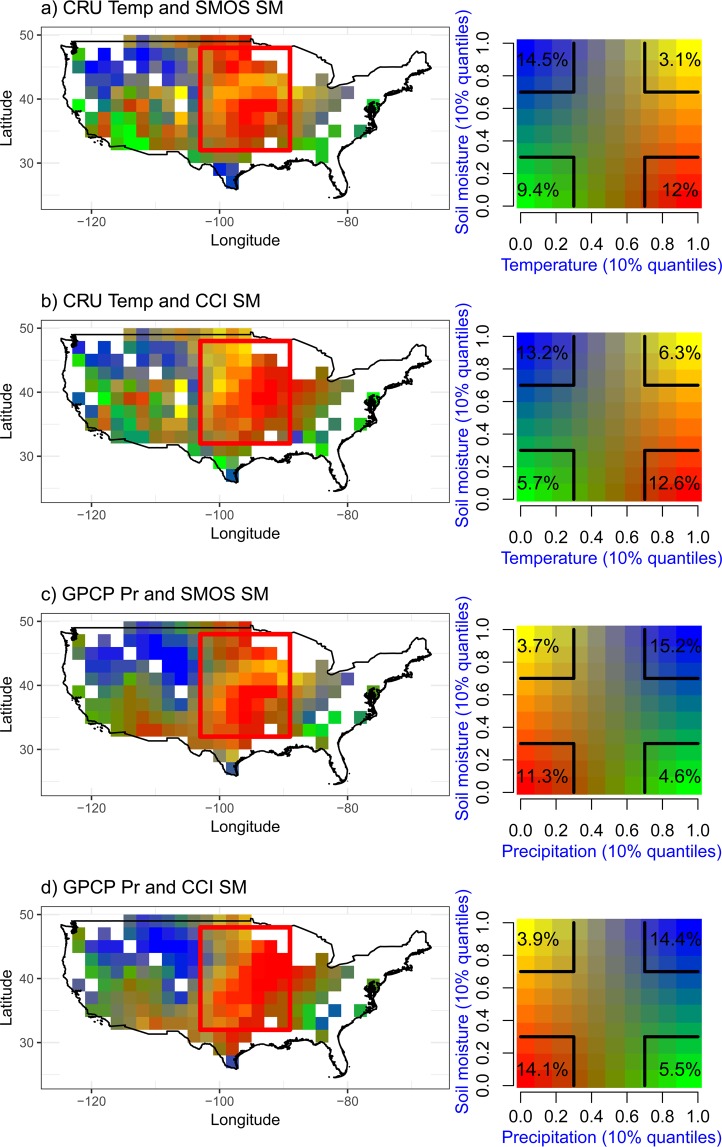
Link between summer soil moisture, air temperature, and precipitation biases. (**a**) Map relating the SMOS SM bias and the CRU air temperature (Temp) bias for the CMIP5 multimodel ensemble. (**b**) Map relating the CCI SM bias and the CRU Temp bias for the CMIP5 multimodel ensemble. (**c**) Map relating the SMOS SM bias and the GPCP precipitation (Pr) bias for the CMIP5 multimodel ensemble. (**d**) Map relating the CCI SM bias and the GPCP Pr bias for the CMIP5 multimodel ensemble. Multimodel reflects results for the CMIP5 ensemble mean. Note that each colour represents a 10% quantile shift (calculated with respect to the spatial histogram of bias results across the CONUS) in both soil moisture and air temperature/precipitation. White regions represent pixels with percentage of forest >60%, strong topography, or frozen soil conditions, which were excluded from the analyses. Each square at the angle of the legends in the right column has a 30% × 30% size with the % of pixels in this square.

In order to better characterize the spatial link between summer biases in air temperature, precipitation and SM, we computed bivariate maps that combine biases in SM (CMIP5 models - SMOS/CCI) and either air temperature (CMIP5 models - CRU) or precipitation (CMIP5 models - GPCP), based on quantile-quantile associations (Fig. 2). The multi-model ensemble (Fig. 2a,b) reveals three main types of bias combinations: (i) a dry and warm bias (in red) mainly over the CGP, (ii) a wet and cold bias (in blue) over north-western CONUS and (iii) a dry and cold bias (in green) in south-western CONUS. Note that, to be more precise, the term “cold” is not really appropriate and we should rather speak of “the smallest warm biases”, since a large majority of the CMIP5 models overestimate mean summer air temperature over the CONUS (Fig. S2). The combination of a dry and cold bias (green areas in Fig. 2a,b) is somewhat surprising. However, several mechanisms can be proposed to explain this. For example, dry soils may lead to excessive albedo or weakened thermal inertia leading to excessive nocturnal cooling during cloudless nights^40^. Conversely, a cold bias can lead to a corresponding dry bias owing to the stabilization of the boundary layer – making it less prone to convection.

**Figure 3:**
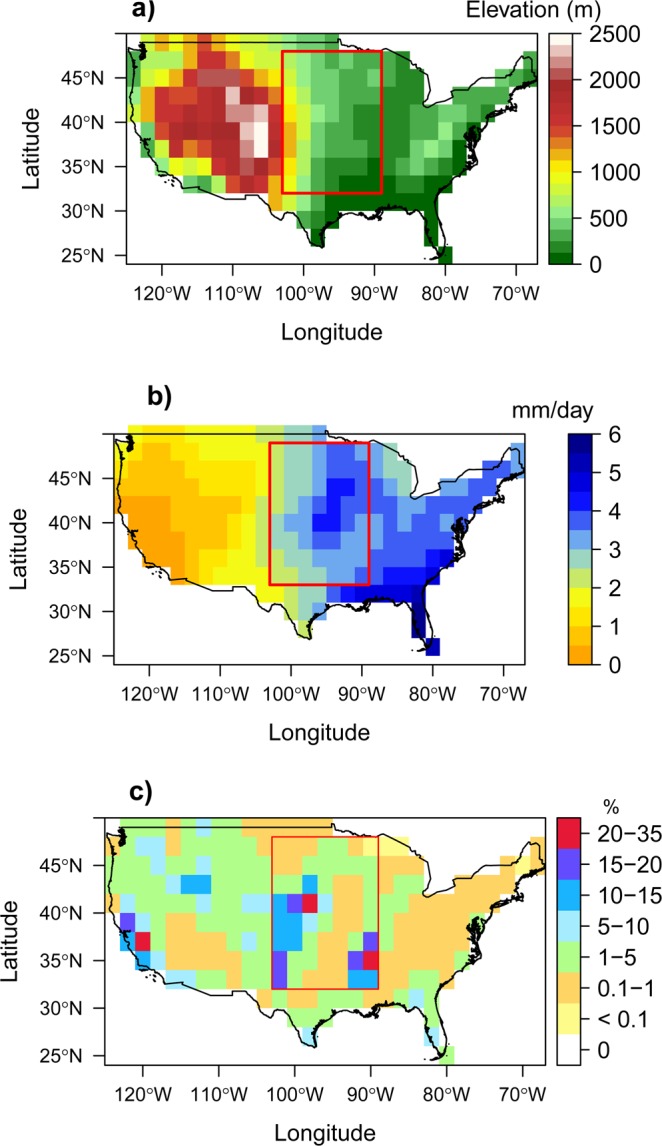
HYDRO 1 K Elevation map of the USA Source http://srtm.csi.cgiar.org/.^74^. Mean GPCP precipitation (mm/day) during the period 1979–2008 considering only JJA months (**b**). Map of areas equipped for irrigation expressed as percentage of total area^56^ (**c**). All maps are re-gridded at the 2° × 2° resolution, to match the bias maps.

Figures S6–7b (Supplementary) display the same maps for each individual CMIP5 model. Despite the variability stemming from inter-model differences, these maps reveal systematic patterns structured by the east-west precipitation gradient and the Rocky Mountains (Fig. 3a,b), which are well-known influential factors on CONUS climate. Interestingly, these patterns are much less systematic when looking at the individual temperature and SM biases present in each of the CMIP5 models (Figs S2–5), suggesting that regional drivers of the CONUS climate have more influence on the relationship between the different types of bias than on the biases themselves. Figures S6–7b also show a few exceptions to the dominance of a warm and dry bias combination in the CGP. These exceptions notably include the two climate models (GISS.E2.R & MRI.AGCM3.2H) characterized by low biases in air temperature and precipitation over CONUS compared to the other models.

The bivariate maps combining the multi-model ensemble SM and precipitation biases in Fig. 2c (using GPCP and SMOS data) and in Fig. 2d (using GPCP and CCI data) show relatively similar spatial patterns to those obtained in Fig. 2a,b (see Supplementary Figs S8–9 for each model separately). In particular, the same three main areas can be distinguished: the CGP region associated with negative SM and precipitation biases (red), north-western CONUS associated with positive SM and precipitation biases (blue) and south-western CONUS (green), where very different responses can be noted depending on the CMIP5 models (red, blue or green colours can be seen in that region for the different models).

Along the Rockies (Fig. 3a; blue areas in Fig. 2a,b), there is a cold bias that can be partly explained by the positive precipitation and SM bias (Fig. 2c,d), via exacerbated ET and/or snow albedo cooling effects. The positive precipitation bias over mountain areas is a classical problem in climate models^41^ and consistent with the categorization of the region as “atmospherically controlled” in Findell and Eltahir^16^. As for the association of a wet and warm bias (yellow areas in Figs 2 and S6–S9), it is quite rare (3 to 6% of the CONUS, depending on the bias association, as quantified on the right panels of Fig. 2) and found mostly in sporadic areas and for a few models over Midwest (northcentral CONUS). Interestingly, the number of grid cells that are warm and dry (red areas in Fig. 2) is very similar whether using SMOS or CCI SM products. Heterogeneous results for the different models are also obtained in eastern CONUS.

### Regional scale analysis over the CGP

**Figure 4:**
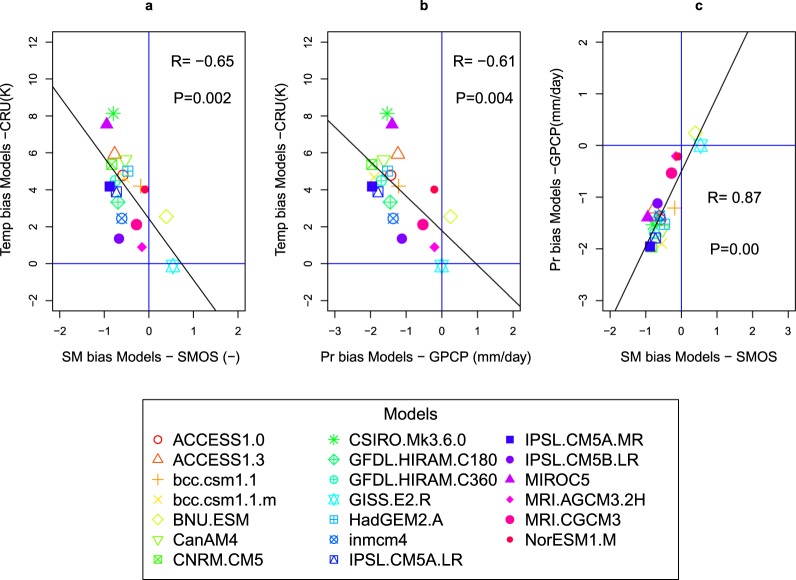
Cross-correlations between the mean biases of the three studied variables over the CGP region among the CMIP5 models. (**a**) Air temperature (Temp) bias (Models-CRU) vs soil moisture (SM) bias (Models-SMOS). (**b**) Temp bias (Models-CRU) vs precipitation (Pr) bias (Models- GPCP). (**c**) Pr bias (Models-GPCP) vs SM bias (Models-SMOS). The inter-model correlation R and p-value are shown on each panel.

We have established a clear link between SM, precipitation and air temperature biases across several large CONUS regions (Figs 2 and S6–9), but the most consistent spatial patterns and the stronger air temperature biases have been obtained over the CGP region (103°W–89°W, 32°N–48°N, displayed as a red box in Figs 1, 2 and 3). Therefore, we now focus on this particular region to better quantify the link between the warm bias found in most models and the negative biases of precipitation and SM. Figure 4a confirms that a large majority of models overestimate air temperature and underestimate SM in the CGP during summer, with a strong negative inter-model correlation between these two biases (R = −0.65; 18 models out of 20 are in the upper-left quadrant), meaning that the warm bias is more pronounced when the dry bias is strong. A strong negative correlation is also found between the biases of precipitation and air temperature among the models, albeit with a slightly smaller correlation coefficient (R = −0.61, the same 18 models are in the upper-left quadrant), which is consistent with the larger variability of atmospheric variables in comparison to surface variables. Eventually, the strongest (positive) inter-model correlation (R = 0.87; the same 18 models are in the lower-left quadrant) are found between the biases in precipitation and SM, in agreement with the fact that precipitation exerts a strong control on SM. The same correlations are obtained when the biases are calculated with respect to the other observational datasets (see Supplementary Fig. S10), which only changes the offset of the scatter plots.

These correlations can arise from two different causal relationships, which are probably entangled:

(i) a negative precipitation bias, likely caused by errors in the general circulation or a convection parameterization, is the driving force and creates a dry surface bias which, in turn, causes a warm bias. For this effect on air temperature, we also have two mechanisms, one is water related (low precipitation can create low SM directly, which reduces the cooling effect of evapotranspiration), and the other is energy related (low precipitation can also induce high downward shortwave radiation which will enhance both the latent and sensible heat fluxes, probably increasing air temperature, and decreasing SM); and
(ii) the land surface is the main cause of the interlinked biases, as low SM reduces evapotranspiration, and therefore warms the lower level of atmosphere and reduces precipitation, with possible enhancement owing to positive SM feedback. The bivariate maps and the correlation analyses (Figs 2 and 4) reveal the link between the spatial patterns in the warm bias of summer air temperature produced by the majority of CMIP5 coupled climate models and dry SM biases, which are significantly correlated to precipitation deficits. In the same region, Cheruy *et al*.^10^ have found a significant negative correlation between the warm bias and the evaporative fraction (EF) of the models (EF = LH/(LH + SH)). Yet the bias in EF was only qualitatively established against one estimate of EF with rather large uncertainty bounds. Our results show, despite observation uncertainties, that the CMIP5 models underestimate SM in the CGP, leading to lower values of EF^24^, which in turn increases (decreases) the sensible heat bias (latent heat) that was already initiated by: i) a failure of climate models to capture heavy rainfalls events associated to mesoscale convective systems^14^; (ii) an excess of surface solar radiation^15,38^; or iii) a failure of climate models to represent the particular type of convective processes (i.e., nocturnal mesoscale convective complexes) that contribute to the majority of summertime rainfall in the CGP^42^. This is in accordance with a widely accepted land-atmosphere coupling mechanism: deficits in precipitation induce dry conditions, thus, favouring less evaporative cooling and higher surface air temperature^14,43–47^. Eventually, at the monthly timescales, based on climatological means, and looking at the model biases on regional average, our results favour a positive feedback between precipitation and air temperature in the CGP.

## Discussion

By analysing the spatial patterns of SM owing to large-scale satellite observations, we demonstrate a specific link between the warm bias and SM bias in CMIP5 models over the CGP, which “closes the loop” by proving the missing land surface link between precipitation and energy fluxes. This link, which has long been suspected in the literature^19,20,22–25,38^, is for the first time shown here on the basis of satellite observations at continental scale, which differs from past studies^15^ based on local-scale *in situ* observations. At a minimum, such satellite SM observations complete our understanding of the forward propagation of rainfall deficiencies through the land surface and into the lower atmosphere (via surface energy fluxes).

Yet, the misrepresentation of SM and related processes could also be a key shortcoming in the modelled climate by means of land-atmosphere coupling. In particular, it has been shown that irrigation has an observable cooling effect at local scale^48–50^, while its effect on precipitation is harder to ascertain. Over the CONUS, recent studies, based on observations, demonstrate a precipitation increase, either locally or downwind of irrigation hotspots^50,51^, and modelling studies report either increases^38^ or decreases^52^ of regional precipitation, with complex teleconnections^53,54^, when accounting for irrigation in coupled land-atmosphere models. Given that irrigation is overlooked by CMIP5 models, a relevant question is whether this missing surface process contributes to the warm and dry biases of CMIP5 models over the CGP region (red box), which includes two of the main three irrigation hotspots of the CONUS (Fig. 3c), i.e. the CGP *stricto sensu* (with withdrawals from the Ogallala aquifer) along the western border of the red box, and the Lower Mississippi valley along its eastern border.

In this framework, we would expect these irrigation hotspots to match regions with negative SM biases in Fig. 1e,f, since the CMIP5 models miss irrigation input. It tends to be the case in the Lower Mississippi valley, but not in the CGP. Interestingly, Kumar *et al*.^55^ assessed several SM retrievals over the CONUS for their irrigation detection skill. All of them, including SMOS, are based on microwave remote sensing and involved in the CCI product used in the present study; and their irrigation detection skill was found to be weak, although better in the Mississippi valley. This “apparent inability” of microwave remote sensing observations to detect irrigation hotspots is partly attributed to the coarse resolution of the retrievals, especially for the passive microwave ones, but our results are suggestive of another explanation, related to regional-scale land-atmosphere coupling over the (extended) CGP (red box).

We speculate that irrigation in this area may not be detected as increased SM values by satellite products, because it locally induces a decrease of precipitation, which feedbacks negatively onto local SM. In terms of model biases, the dry bias expected from missing irrigation would be offset by a positive precipitation bias (by missing irrigation-induced decrease of precipitation). Another explanation might be that the “extra” water provided by irrigation is quickly used by the vegetation and not visible at the monthly to seasonal scale. Both explanations remain consistent with the warm bias found over the CGP irrigation hotspots, since evaporation would miss in models overlooking irrigation; this missing evaporation could then contribute to the dry (for both precipitation and SM) and warm biases downwind of the CGP, corresponding to the red areas in the central south part of the red box of Fig. 2a,b.

## Conclusion

This research is the first one analysing the link between spatial patterns of SM biases and those of precipitation and air temperature in the context of CMIP5 models over the CONUS. The results confirm that SM, as a key driver of the water and energy fluxes at the land/atmosphere interface, must be accounted for to better understand the deficiencies of climate models. In combination with previous studies, our results also support the argument that land-atmosphere feedbacks over the extended CGP are not only local but have a regional dimension, owing to the atmospheric circulation. This could explain why the strongest associations between the dry and warm biases (dark red in Fig. 2) are found between the irrigation hotspots, while these hotspots rather correspond to warm/wet bias associations (yellow-shaded areas in Fig. 2).

Further work is required to ascertain these assumptions over the CONUS, and potentially generalize them in other regions of strong land-atmosphere coupling^21^, frequently associated to hotspots of irrigation^56^, where present-day biases can cast doubt on the magnitude of climate change response^7,8,44^. This calls for continued and improved large-scale SM satellite observations on the one hand, and for numerical experiments tailored to better understand the role of SM and irrigation in climate models on the other hand, as planned in the framework of CMIP6. Two CMIP6-Endorsed Model Intercomparison Projects (MIPs) are particularly relevant, namely LUMIP (Land Use MIP^57^), and LS3MIP (Land Surface, Snow and Soil moisture MIP^58^), where the effect of irrigation and SM nudging, respectively, will be explored retrospectively and in future projections across a wide number of climate models.

## Methods

In this study, we compared climate model outputs for 2-m near surface air temperature, precipitation, and SM to corresponding gridded observational data. This comparison is based on climatological means for summer (JJA) over CONUS and CGP, and involves correlations and simple statistics like quantile-quantile associations (spatially over CONUS), and inter-model correlations (based on spatial averages over CGP).

### Gridded observations

#### 2-m near surface air temperature data

We used two data sets based on *in situ* measurements of near surface temperature (referred to as air temperature):

(i) the gridded time-series Climate Research Unit (CRU) air temperature dataset Version 4.2^59^ produced by the Climate Research Unit at the University of East Anglia (U.K.) on a global scale (only land) with monthly resolution and a spatial resolution of 0.5° × 0.5°, available from 1901 to 2015; and
(ii) the University Of Delaware gridded air temperature data set^60^ provided on a global scale (only land) with monthly resolution and a spatial resolution of 0.5° × 0.5°, available from 1901 to 2014.

#### Precipitation data

As a precipitation reference, we used two different kinds of products, which provide very close climatological means in summer over the USA (Fig. 1c,d):

(i) the gridded time-series Climate Research Unit (CRU) precipitation dataset Version 3.4 (Harris *et al*.^59^) produced by the Climate Research Unit at the University of East Anglia (U.K.) on a global scale (only land) with monthly resolution and a spatial resolution of 0.5° × 0.5° from 1901 to 2015. This data set is entirely based on rain-gauge data (11,800 worldwide); and
(ii) the Global Precipitation Climatology Project (GPCP) precipitation datasets (Version 2.3) provided on a global scale with monthly resolution and spatial resolution of 2.5° × 2.5° from 1979 to present^61^. The GPCP monthly precipitation datasets are produced by merging observations from satellites, rain gauge stations, and sounding observations.

#### Soil moisture data

As SM reference, we used two satellite-derived products, both providing retrievals of surface SM (top 5 cm):

The most recent re-processed SMOS-IC SM product retrieved from SMOS Satellite brightness temperature (TB) observations. The SMOS satellite was launched in 2009 by the European Space Agency (ESA) to monitor SM and sea surface salinity at the global scale^62,63^. SMOS-IC SM was recently developed by INRA (Institut National de la Recherche Agronomique) in collaboration with CESBIO (Centre d’Etudes Spatiales de la BIOsphère). The SMOS-IC SM product differs from the operational SMOS Level 3 SM product (SMOSL3) in three main ways: (i) SMOS-IC is based on SMOS Level 3 TB data and it is independent from auxiliary model products, (ii) specific filters were introduced to select the TB data used in the SM retrievals, and (iii) a new calibration of the soil roughness parameters and vegetation parameters was applied in the forward L-MEB (L-band Microwave Emission of the Biosphere) model. SMOS-IC provides SM (in m^3^/m^3^) for the first 5 cm centimetres of the soil and is available for the period 2010–2016 on a daily basis and at a spatial resolution of 25 km. More details on the SMOS-IC data can be found in Fernandez-Moran *et al*.^64^.

(i) The ESA CCI combined (version 03.2) SM product was generated by combining different active and passive microwave SM retrievals from different sensors: ERS, ASCAT (Metop-A and Metop-B), SSMR, TMI, AMSR-E, SSM/I, SMOS, AMSR2, and WindSat^34,65–67^. It should be noted that this CCI version included more recent sensors (e.g., SMOS and AMSR2) that were not considered in the previous versions of the CCI SM products. Hence, a significant increase in the scientific value of the products is expected. It is worth noting that the SMOS SM product included in the CCI was derived using the LPRM algorithm which is different from the SMOS-IC products used in this study. The CCI SM product is provided as daily SM (in m^3^/m^3^) for the first 5 cm of the soil column and is available for the 1978–2015 period with a spatial resolution of 0.25° × 0.25°.

Both the SMOS (the version used here and earlier versions) and CCI SM retrievals have been extensively evaluated at both local and global scales^36,63,68,69^. Here, we show an example of the performance of both CCI and SMOS products against *in situ* SM observations from the Atmospheric Radiation Measurement (ARM) dataset, which includes 19 stations from the International Soil Moisture Network (ISMN; https://ismn.geo.tuwien.ac.at/), all situated in the CGP region. Each *in situ* station was independently collocated to the closest grid points of SMOS and CCI based on its longitude and latitude coordinates and the corresponding CCI and SMOS SM retrievals were compared to the ARM time series on a daily basis within the period 2010–2017. Then the statistics of the inter-comparison were computed based on results obtained over each single pixel for both SMOS and CCI. The resulting, Pearson correlation, and normalized Root-Mean-Square Error and standard deviation, are summarized in a Taylor diagram^70^ (see Supplementary Fig. S11), showing that SMOS and CCI share fair and comparable performance with respect to the overall ARM dataset: most correlation values range between 0.6 and 0.8, and standard deviations tend to be overestimated. This error may be due to differences in sampling depth between *in situ* measurements and remote sensing products), but it is reduced by the normalization procedure aimed at enhancing SM comparability between remote sensing and climate models (details below).

It should be noted that there are some uncertainties in remote sensing retrieved SM originating from the sensor type, scales, calibration, the observation geometry, parameters/auxiliary fields used in the SM inversion algorithms, sampling depth mismatch between models and satellite-based SM retrievals, and/or uncertainties introduced by underlying assumptions, which should not be neglected when applying remote sensing observations in model evaluation.

#### CMIP5 simulations

We focused on so-called AMIP simulations (historical land-atmosphere simulations with prescribed sea surface temperatures over 1979–2008) of the CMIP5 project and selected 20 models providing the following three output variables: 1) TAS: 2-m near surface air temperature (in K); 2) Pr: total precipitation (in kg m^−2^ s^−1^); 3) mrsos: moisture in upper portion of soil column (10 cm; in kg/m^2^). It is often argued that SM variability varies with soil depth^36,71^, but it was not possible to extract the top 5-cm SM for a large enough number of models. Yet, a recent comparison of *in situ* and modelled SM profiles showed very similar monthly mean SM values at 5 and 10 cm^72^, while several observational studies reviewed in Gruber *et al*.^73^ reported a good correlation of SM measured at various depths, leading to persistent patterns of SM. Eventually, to make the 10-cm modelled SM as comparable as possible to the 5-cm remotely sensed SM, we relied on both long-term averaging (JJA) and a normalization procedure^71^, as described below. More details about the 20 models used in this study can be found in Table S1 (Supplementary).

#### Processing of the data sets

Given that all data (observations and simulations) were provided in different spatial resolutions, all datasets used in this study were re-gridded to a 2° × 2° resolution following Cheruy *et al*.^10^. The period used for this analysis is 1978–2008 for all datasets with the exception of the SMOS dataset which is only available from 2010 to 2016. By using SMOS in this comparison, we are making an implicit assumption of first-order stationarity in the SM time series (i.e., the mean in 2010–2016 should match the mean in 1979–2008). Since mid-latitudinal summer drying is a common climate change signal predicted by climate models, we examined if this assumption could lead to misleading conclusions. This was done by using CCI data over different periods: 1978–2008, 1987–2008, 2010–2016 (used period for SMOS) and confirming that resulting SM biases were consistent across these three periods (not shown here). Pixels with percentage of forest >60%, strong topography, or frozen soil conditions were excluded from the analyses.

For the precipitation and air temperature variables, the bias of summer air temperature (JJA) was computed on each 2° × 2° pixel between the simulations and the observations over the 1979–2008 period. In addition, the CONUS was selected in this study to avoid the negative impact of RFI (Radio Frequency Interference) on the SMOS SM retrievals, as other regions with comparable warm biases (i.e., Europe and India) are partially contaminated with RFI and there is almost no RFI over the CONUS. For SM, both the simulations and the observations were normalized spatially (SMn) before computing the local biases:

1. sampling of the temporal mean and standard deviation in each 2° × 2° pixel for each dataset over 1978–2008, 2010–2016, and 1979–2008 considering all months for CCI, SMOS-IC, and CMIP5, respectively. This step produces two maps of temporal mean and standard deviation for each dataset separately;
2. sampling of the spatial mean (here referred to as M) of the temporal mean and standard deviation (here referred to as SD) maps produced in step 1 over the CONUS. The output of the step is presented in Table S2 (Supplementary); and
3. normalization of the original datasets *SM*_*i*_ using the values computed in step 2, shown in Table S2 (Supplementary), as follows:

1 $$SMni=\frac{S{M}_{i}-M}{SD}$$

Next, the normalized data produced in the previous steps were averaged considering only summer (JJA months) for the remotely sensed SM retrievals and the CMIP5 SM simulations. Note that the mean of the normalized data is non-zero because M is calculated using all months (and spatially averaged) while the normalized data is computed only over JJA (and not over the full year). Finally, the difference in the overall mean of the normalized data between the CMIP5 models simulations and the remotely sensed SM products (SMOS-IC and CCI) was computed.

## Supplementary information

SUPPLEMENTARY INFO

## Data Availability

SMOS-IC SM datasets are publicly available at CATDS (Centre Aval de Traitement des Données SMOS): https://www.catds.fr/Products/Available-products-from-CEC-SM/SMOS-IC. CCI SM datasets are freely available upon registration at http://www.esa-soilmoisture-cci.org/. Monthly CRU air temperature and precipitation are publicly available at http://www.cru.uea.ac.uk/. Monthly Willmott air temperature observations are freely available at https://www.esrl.noaa.gov/psd/data/gridded/data.UDel_AirT_Precip.html. Monthly GPCP precipitation datasets are freely available at https://www.esrl.noaa.gov/psd/data/gridded/data.gpcp.html. Monthly CMIP5 air temperature, SM, and precipitation simulations are freely available from https://cmip.llnl.gov/cmip5/data_portal.html. HYDRO 1K Elevation Derivative Data are available from the U.S. Geological Survey and can be freely downloaded from: https://lta.cr.usgs.gov/HYDRO1K.
