# Supplementary material for: Satellite-based soil moisture provides missing link between summertime precipitation and surface temperature biases in CMIP5 simulations over conterminous United States
Source: Sci Rep. 2019 Feb 7;9:1657. doi: 10.1038/s41598-018-38309-5 (PMC6367487; doi:10.1038/s41598-018-38309-5)
Supplement: Supplementary file 1 — SUPPLEMENTARY INFO [file 41598_2018_38309_MOESM1_ESM.docx]

**Supplementary Information “**Satellite-based soil moisture provides missing link between summertime precipitation and surface temperature biases in CMIP5 simulations over conterminous United States**”**

A. Al-Yaari^1*^, A. Ducharne^2^, F. Cheruy^3^, W. T. Crow^4^,J.-P. Wigneron^1^

# (1) INRA, UMR 1391 ISPA, Villenave d’Ornon, France

# (2) Unité Mixte de Recherche METIS, IPSL, Sorbonne Université, CNRS, EPHE, Paris, France

# (3) Laboratoire de Météorologie Dynamique, IPSL, CNRS, Sorbonne Universités, Paris, France

# (4) Hydrology and Remote Sensing Lab, USDA ARS, Beltsville, MD, USA

*Correspondence to [amen.al-yaari@inra.fr]

**Tables**

| **Model(s)** | **Host Institute** | **Resolution (atmosphere)** | **References** |
| --- | --- | --- | --- |
| **ACCESS1.0 ACCESS1.3** | CSIRO (Commonwealth Scientific and Industrial Research Organisation, Australia) and BOM (Bureau of Meteorology, Australia) | 1.9° × 1.3°  L38 | Bi et al. [^1^](#_ENREF_1) |
| **BCC-CSM1.1-M**  **BCC-CSM1.1** | Beijing Climate Center, China Meteorological Administration, China | 1.1° × 1.1°,L26  2.8° × 2.8°, L26; | Wu et al. [^2^](#_ENREF_2) |
| **BNU-ESM** | College of Global Change and Earth System Science (GCESS), BNU, Beijing, China | 2.8° × 2.8°  L26 | Ji et al. [^3^](#_ENREF_3) |
| **CanAM4** | Canadian Center for Atmospheric Research, Canada | 2.8° × 2.8°  L35 | Arora et al. [^4^](#_ENREF_4) |
| **CNRM-CM5** | Centre National de Recherches Météorologiques (CNRM), Météo-France and Centre Européen de Recherches et de Formation Avancée en Calcul Scientifique (CERFACS), France | 1.4° × 1.4°  L31 | Voldoire et al. [^5^](#_ENREF_5) |
| **CSIRO-Mk3.6.0** | Australian Commonwealth Scientific and Industrial Research Organization (CSIRO) Marine and Atmospheric Research, Queensland Climate Change Centre of Excellence (QCCCE), Australia | 1.9° × 1.9°  L18 | Rotstayn et al. [^6^](#_ENREF_6) |
| **GFDL-HIRAM-C360; GFDL-HIRAM-C180;** | Geophysical Fluid Dynamics Laboratory (NOAA GFDL), United States | 2.5° × 2.5° | Donner et al. [^7^](#_ENREF_7); http://nomads.gfdl.noaa.gov/ |
| **GISS-E2-R** | Goddard Institute for Space Studies (NASA/GISS),United States | 2.5° × 2°  L40 | Schmidt et al. [^8^](#_ENREF_8) |
| **HadGEM2-A** | Met Office Hadley Centre,United Kingdom | 1.9° × 1.3° | Collins et al. [^9^](#_ENREF_9) |
| **INM-CM4** | Institute for Numerical Mathematics (INM), Russia | 2° × 1.5°  L21 | Volodin et al. [^10^](#_ENREF_10) |
| **IPSL-CM5A-LR; IPSL-CM5A-MR; IPSL-CM5B-LR** | Institut Pierre Simon Laplace (IPSL), France | 3.8° × 1.9°;  2.5° × 0.6°;  3.8° × 1.9°  L39 | Dufresne et al. [^11^](#_ENREF_11) |
| **MIROC5** | Japan Agency for Marine-Earth Science and Technology (JAMSTEC), Atmosphere and Ocean Research Institute (AORI), University of Tokyo and National Institute for Environmental Studies (NIES),Japan | 1.4° × 1.4°  L40 | Watanabe et al. [^12^](#_ENREF_12) |
| **MRI-CGCM3; MRI-AGCM3** | Meteorological Research Institute (MRI);Japan | 1.1° × 1.1°  L48 | Yukimoto et al. [^13^](#_ENREF_13) |
| **NorESM1-M** | Norwegian Climate Centre; Norway | 2.5° × 1.9°  L26 | Bentsen et al. [^14^](#_ENREF_14) |

**Table S1.** CMIP 5 models information. Source:[^15^](#_ENREF_15)

**Table S2.** The spatial mean and standard values used for normalizing the soil moisture datasets.

| **datasets** | **Spatial Mean**  **(M)** | **Spatial standard deviation**  **(SD)** |
| --- | --- | --- |
| **Models** | m^3^/m^3^ | m^3^/m^3^ |
| ACCESS1.0 | 0.164 | 0.073 |
| ACCESS1.3 | 0.203 | 0.055 |
| BCC-CSM1.1 | 0.152 | 0.031 |
| BCC-CSM1.1-M | 0.150 | 0.032 |
| BNU-ESM | 0.174 | 0.037 |
| CanAM4 | 0.158 | 0.093 |
| CNRM-CM5 | 0.015 | 0.005 |
| CSIRO-Mk3.6.0 | 0.244 | 0.046 |
| GFDL-HIRAM-C180 | 0.202 | 0.037 |
| GFDL-HIRAM-C360 | 0.198 | 0.035 |
| GISS-E2-R | 0.242 | 0.054 |
| HadGEM2-A | 0.159 | 0.068 |
| INM-CM4 | 0.253 | 0.080 |
| IPSL-CM5A-LR | 0.075 | 0.037 |
| IPSL-CM5A-MR | 0.072 | 0.038 |
| IPSL-CM5B-LR | 0.084 | 0.031 |
| MIROC5 | 0.206 | 0.049 |
| MRI-AGCM3 | 0.201 | 0.048 |
| MRI-CGCM3 | 0.239 | 0.056 |
| NorESM1-M | 0.212 | 0.041 |
| **Observations** |  |  |
| SMOS-IC | 0.133 | 0.067 |
| CCI | 0.194 | 0.047 |

**Figures**





Figure S1. Scatter plots showing the mean bias between multi-model CMIP5 and observations of each pair of the different variables: (a) CRU and Willmott temperature bias (TAS), (b) CRU and GPCP precipitation bias (pr; mm), and (c) SMOS and CCI spatially normalized soil moisture (SM).


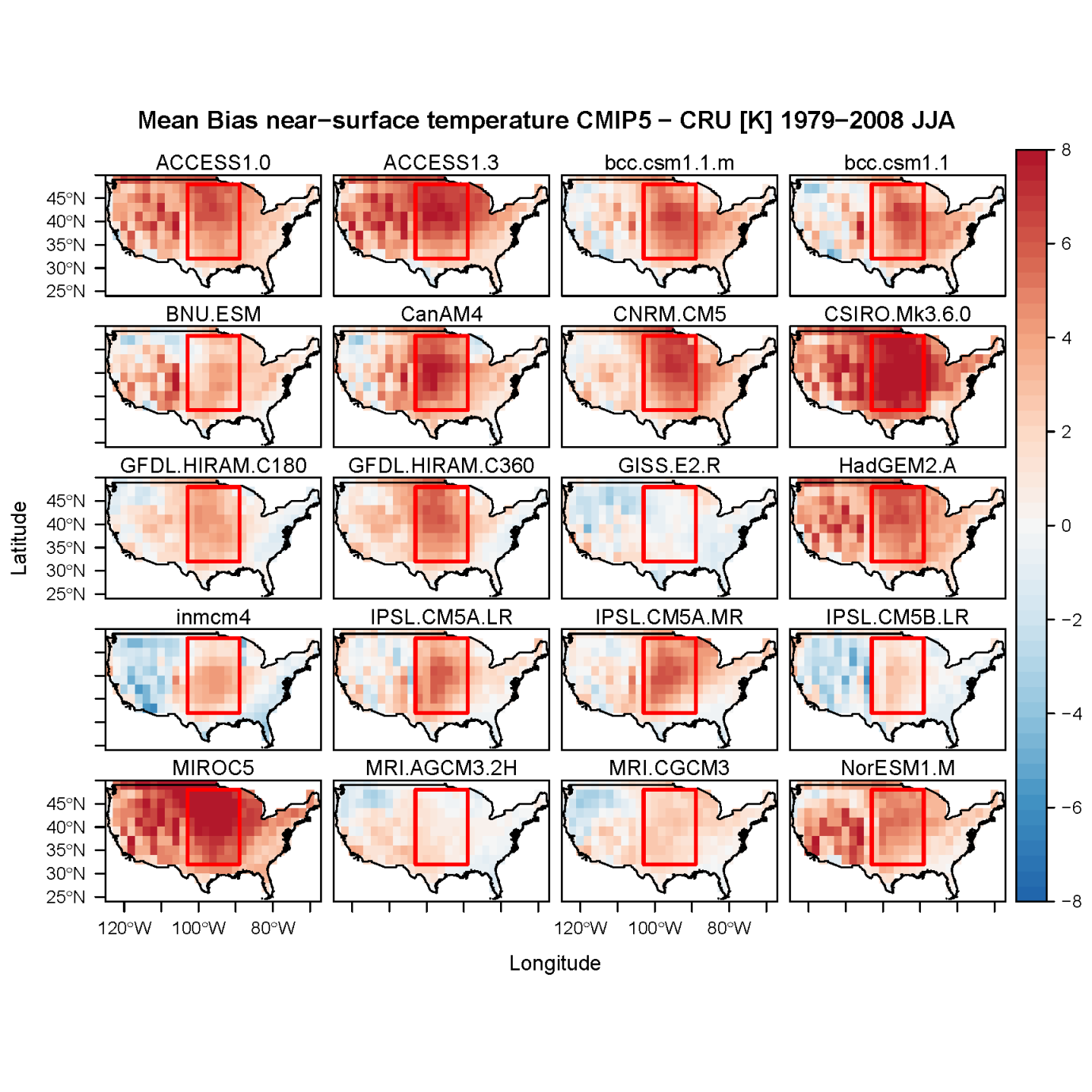


*Figure S2. Mean bias of summer air temperature (K) CMIP5 models – CRU datasets (1979-2008; JJA).*


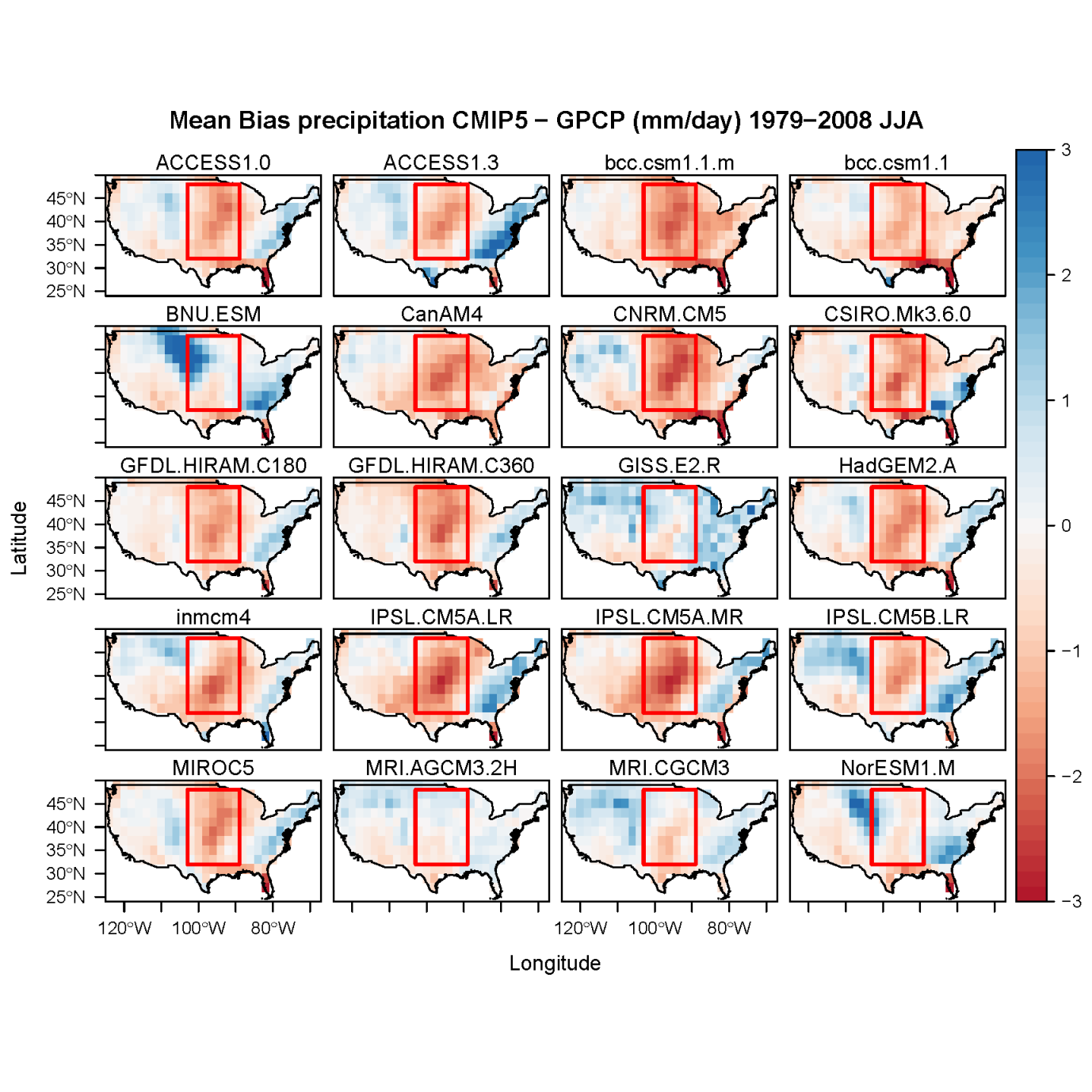


*Figure S3. Mean bias of summer precipitation (mm/day) CMIP5 models – GPCP data (1979-2008; JJA).*


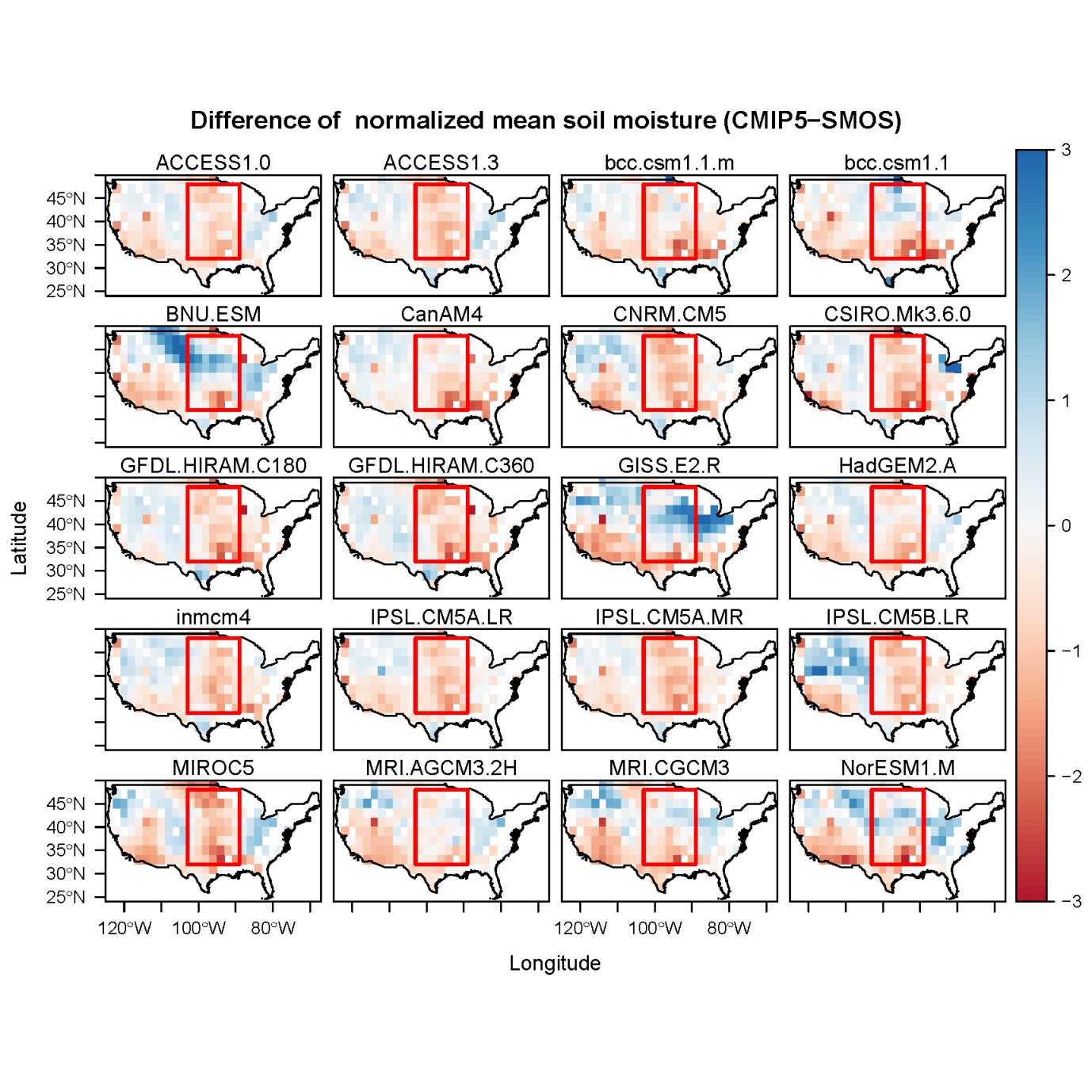


Figure S4. Difference between mean normalized CMIP5 (1978-2008; JJA) and SMOS-IC (2010-2016; JJA) soil moisture data.


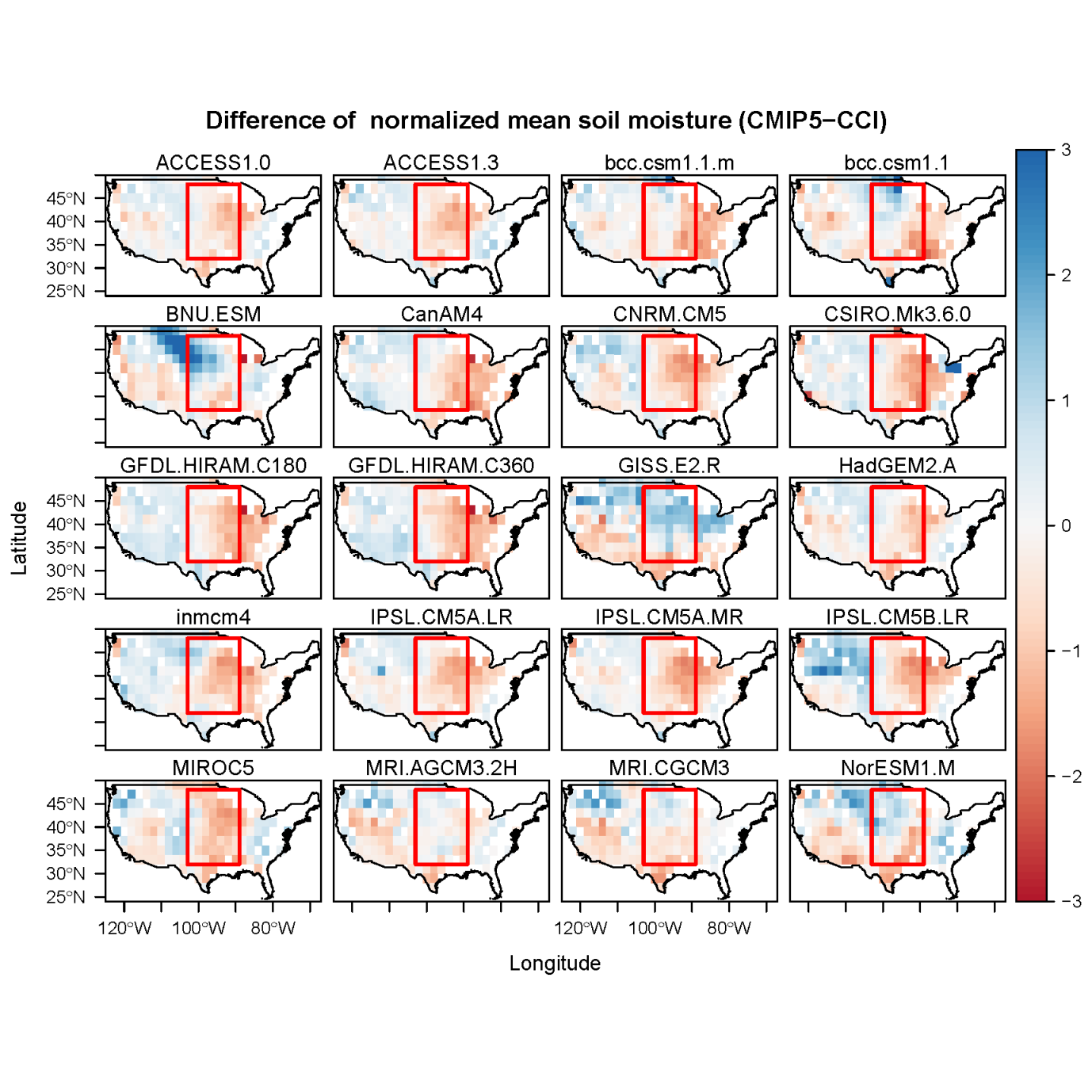


Figure S5. Difference between mean normalized CMIP5 (1978-2008; JJA) and CCI (2003-2015; JJA) soil moisture data.


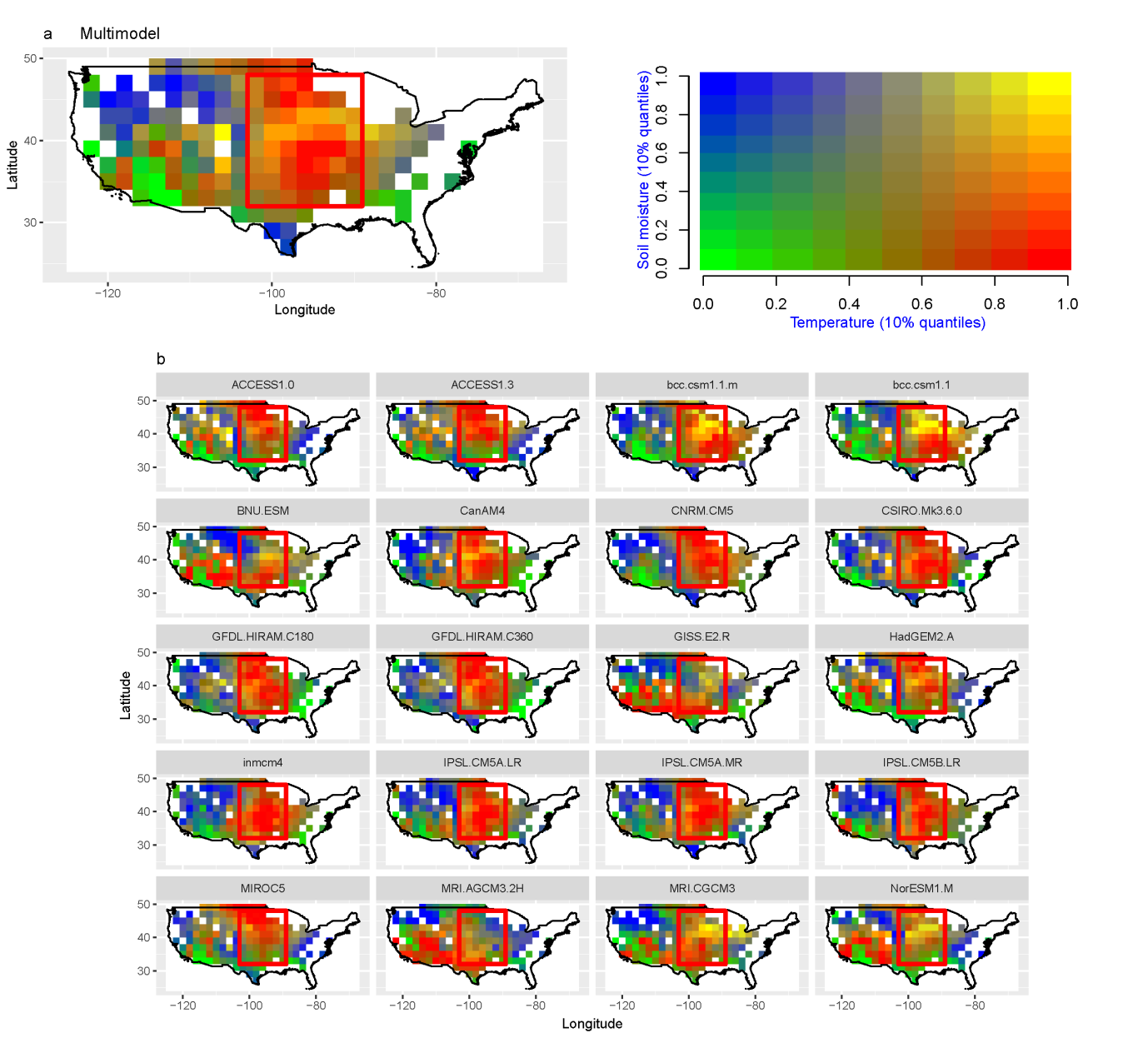


Figure S6. Link between bias summer air temperature and SMOS soil moisture. a, map relating the SMOS SM bias and the CRU air temperature bias for the CMIP5 multimodel ensemble. Multimodel results reflect results for the CMIP5 ensemble mean. b, maps relating the SMOS soil moisture bias and the CRU air temperature bias for the 20 CMIP5 models. Note that each colour represents a 10% quantile shift (calculated with respect to the spatial histogram of bias results across the CONUS) in both soil moisture and air temperature.


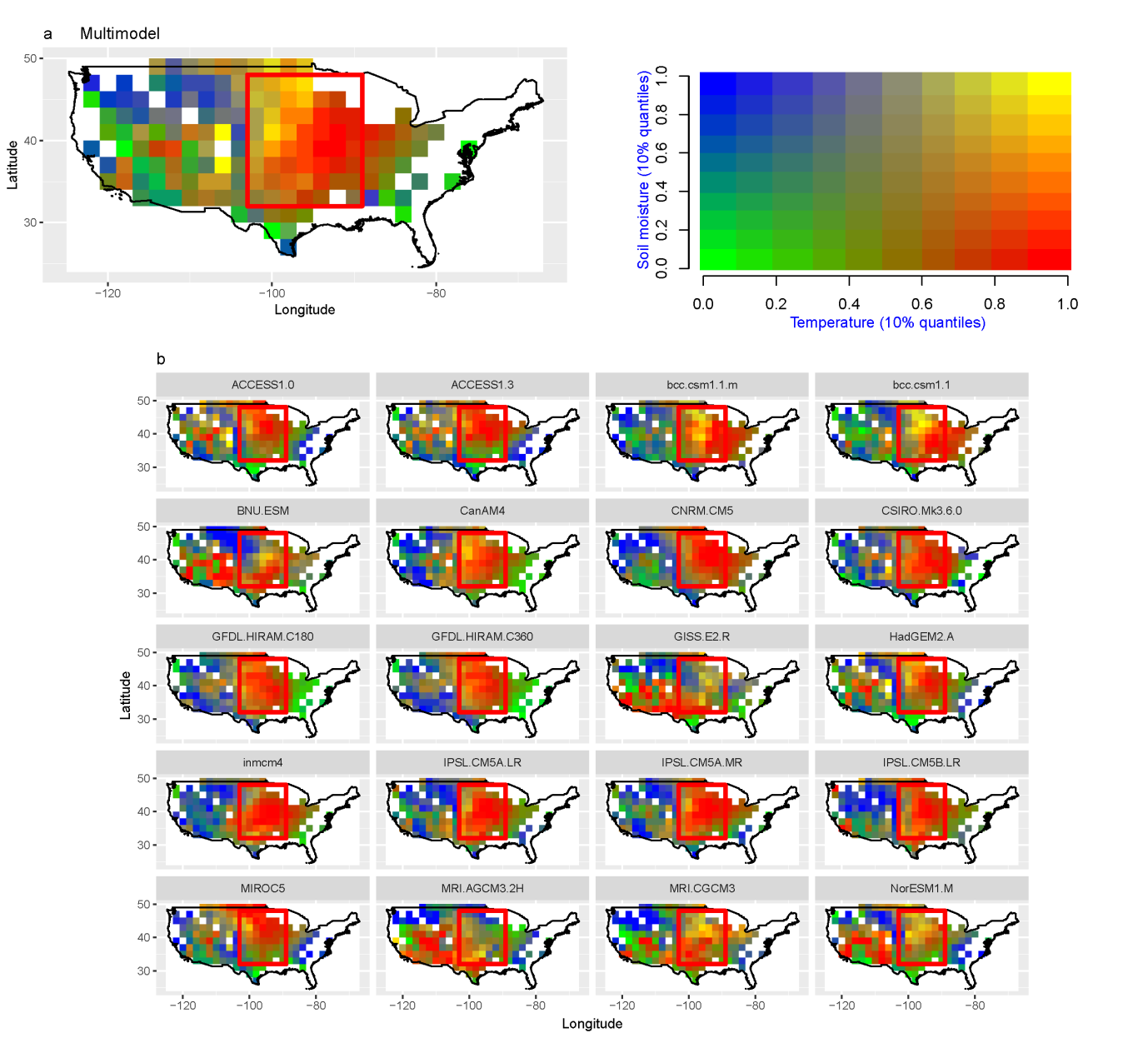


Figure S7. Link between biases in CRU summer air temperature and CCI soil moisture. a, maps relating the CCI soil moisture bias and the CRU air temperature bias for the 20 CMIP5 models. b, map relating the CCI soil moisture bias and the CRU air temperature bias for CMIP5 multimodel ensemble. Note that each colour represents a 10% quantile shift in both soil moisture and air temperature.


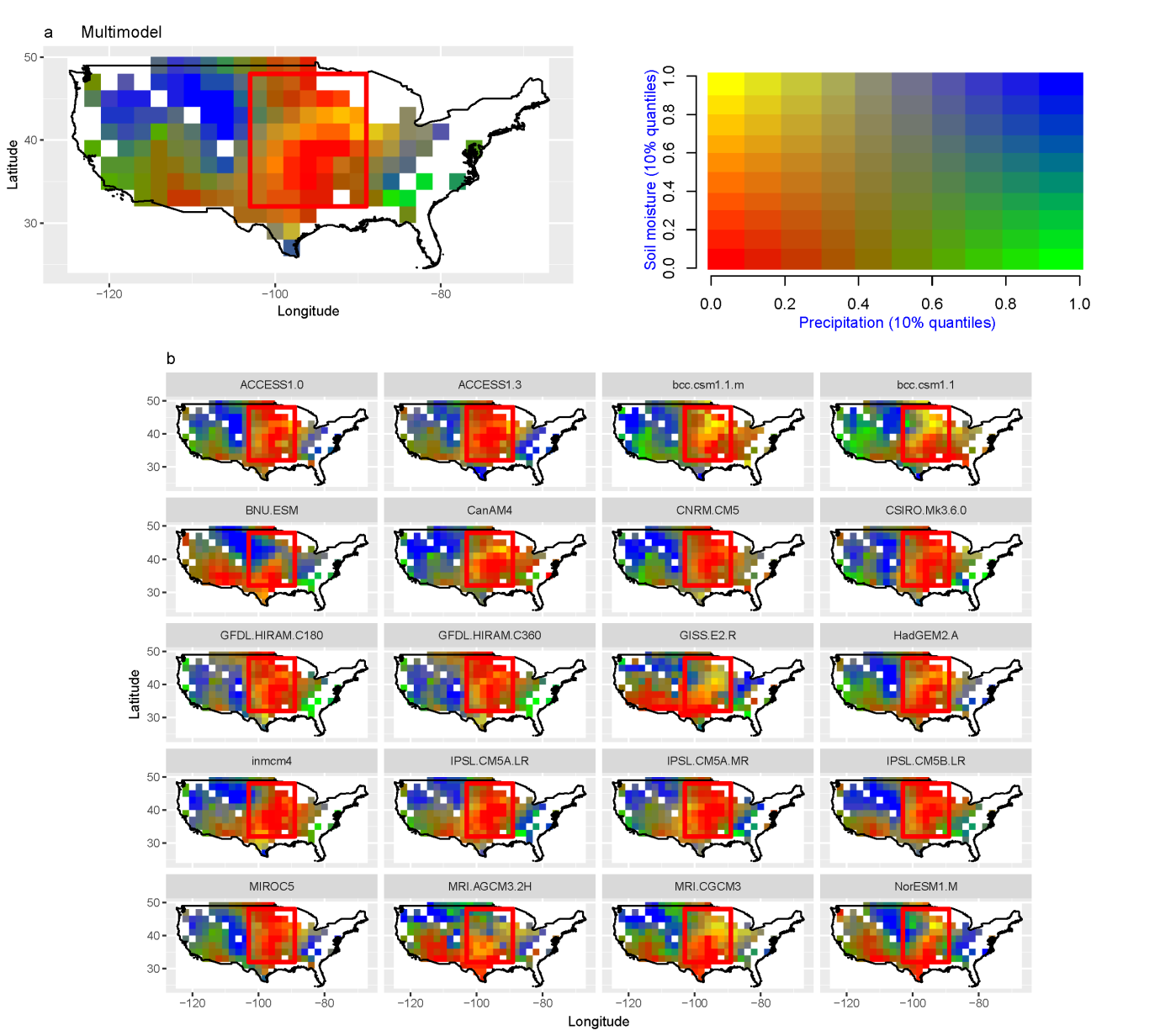


Figure S8. Link between biases in SMOS soil moisture and GPCP precipitation. a, maps relating the SMOS soil moisture bias and the GPCP precipitation bias for the 20 CMIP5 models. b, map relating the SMOS soil moisture bias and the GPCP precipitation bias for CMIP5 multimodel ensemble. Note that each colour represents a 10% quantile shift in both soil moisture and precipitation.


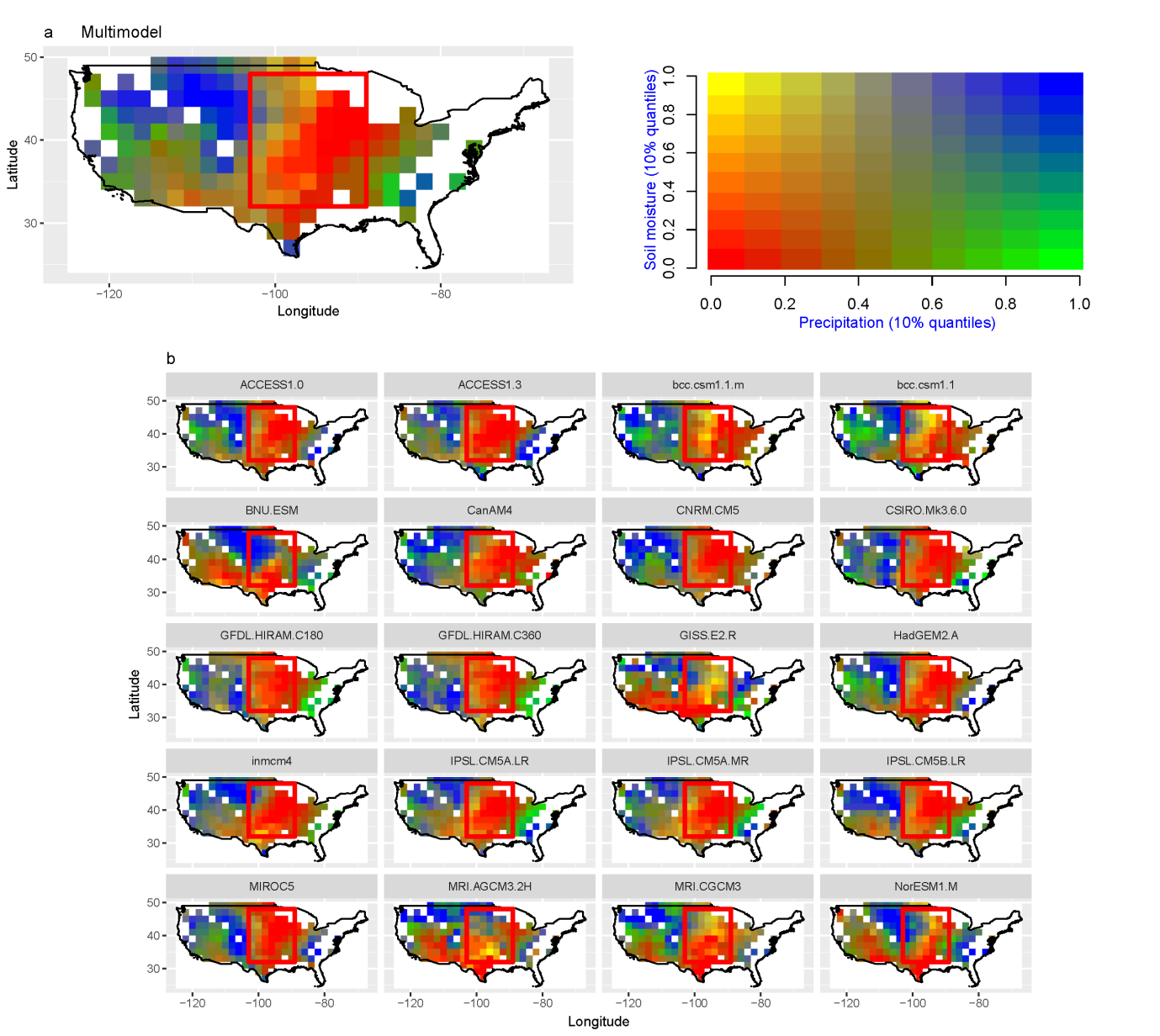


Figure S9. Link between biases in CCI soil moisture and GPCP precipitation. a, maps relating the CCI soil moisture bias and the GPCP precipitation bias for the 20 CMIP5 models. b, map relating the CCI soil moisture bias and the GPCP precipitation bias for CMIP5 multimodel ensemble. Note that each colour represents a 10% quantile shift in both soil moisture and precipitation.

**
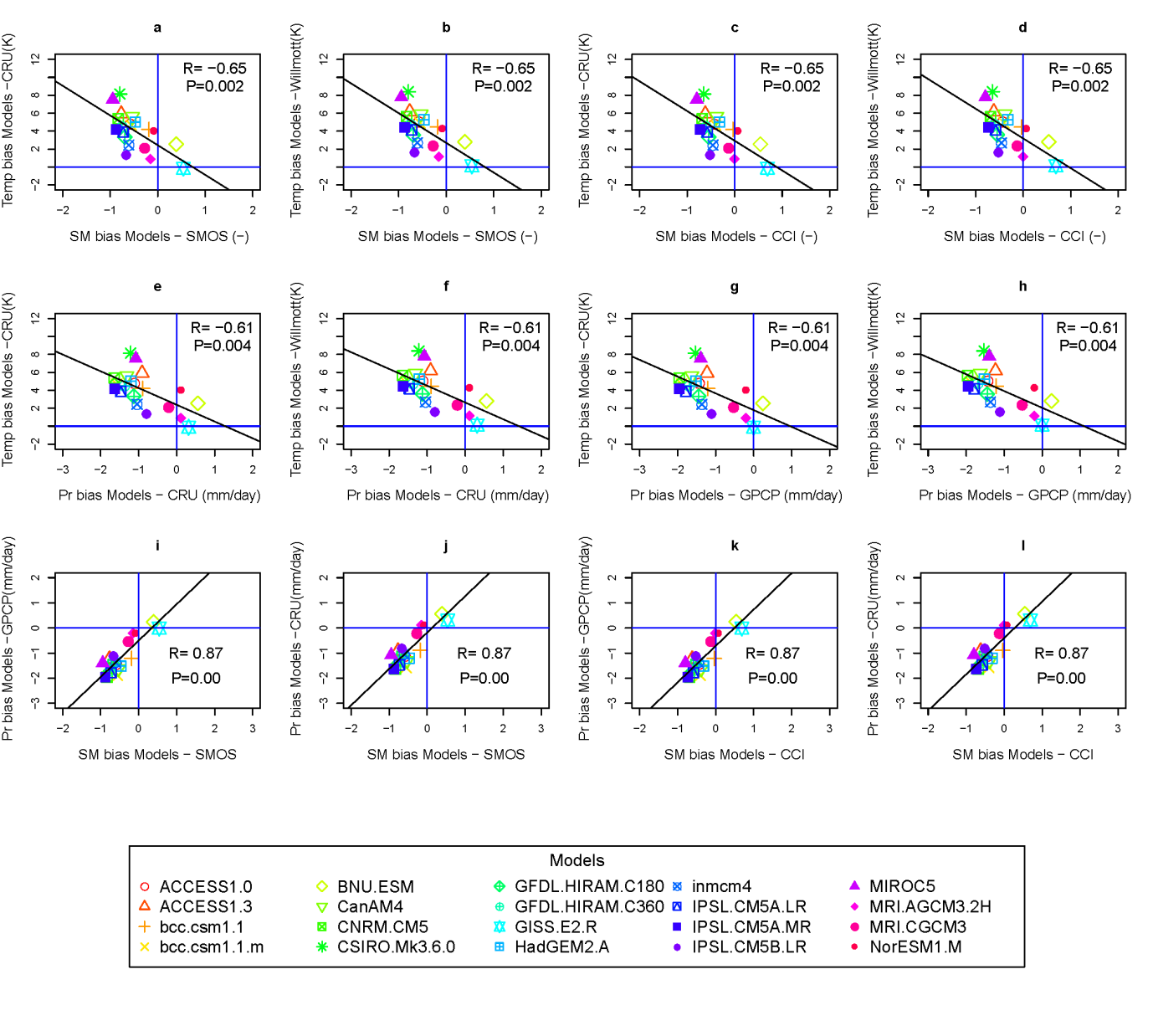
**

Figure S10. Cross-correlations between of the mean biases of the three studied variables over the CGP among the CMIP5 models. Top row: air temperature (Temp) bias vs soil moisture (SM) bias. a, Temp bias (Models-CRU) vs SM bias (Models-SMOS-IC). b, Temp bias (Models-Willmott) vs SM bias (Models-SMOS-IC). c, Temp bias (Models-CRU) vs SM bias (Models-CCI). d, Temp bias (Models-Willmott) vs SM bias (Models-CCI). Middle row: Temp bias vs precipitation (P) bias e, Temp bias (Models-CRU) vs Pr bias (Models- CRU). f, Temp bias (Models-Willmott) vs Pr bias (Models- CRU). g, Temp bias (Models-CRU) vs Pr bias (Models- GPCP). h, Temp bias (Models-Willmott) vs Pr bias (Models- GPCP). Bottom row: Pr bias vs SM bias: i, Pr bias (Models-GPCP) vs SM bias (Models-SMOS-IC). j, Pr bias (Models-CRU) vs SM bias (Models-SMOS-IC). k, Pr bias (Models-GPCP) vs SM bias (Models-CCI). l, Pr bias (Models-CRU) vs SM bias (Models-CCI). The inter-model correlation R and p-value are shown on each panel.

**
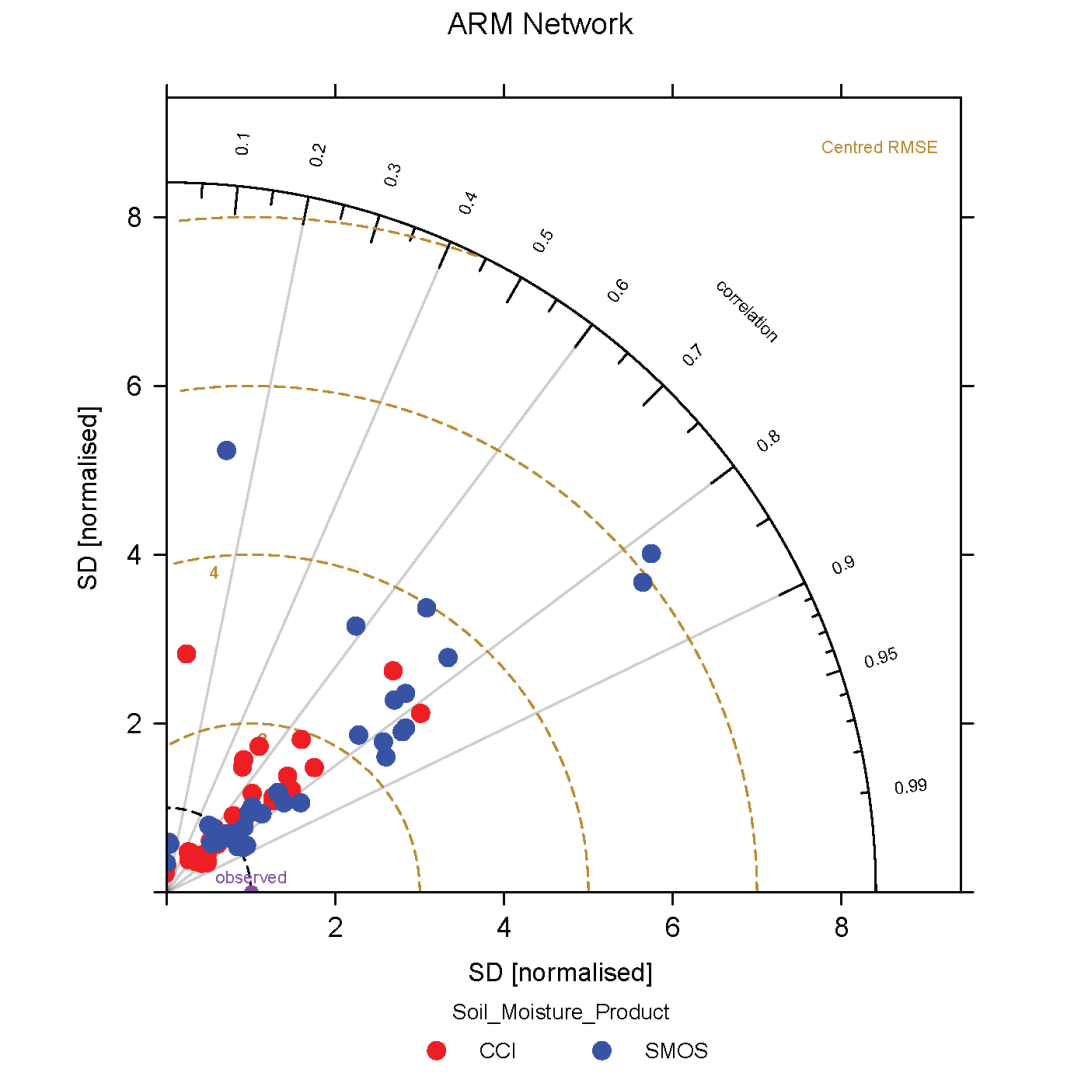
**

Figure S11. Taylor diagram comparing CCI and SMOS soil moisture retrievals to ARM in situ observations using daily values available within the period 2010-2017. Note that the centred RMSE and standard deviation (SD) are normalized by the standard deviation of the observations.

**References**

1 Bi, D. *et al.* The ACCESS coupled model: description, control climate and evaluation. *Aust. Meteorol. Oceanogr. J.* **63**, 41-64 (2013).

2 Wu, T. A mass-flux cumulus parameterization scheme for large-scale models: description and test with observations. *Climate Dynamics* **38**, 725-744, doi:10.1007/s00382-011-0995-3 (2012).

3 Ji, D. *et al.* Description and basic evaluation of Beijing Normal University Earth System Model (BNU-ESM) version 1. *Geosci. Model Dev.* **7**, 2039-2064, doi:10.5194/gmd-7-2039-2014 (2014).

4 Arora, V. K. *et al.* Carbon emission limits required to satisfy future representative concentration pathways of greenhouse gases. *Geophysical Research Letters* **38**, n/a-n/a, doi:10.1029/2010gl046270 (2011).

5 Voldoire, A. *et al.* The CNRM-CM5.1 global climate model: description and basic evaluation. *Climate Dynamics* **40**, 2091-2121, doi:10.1007/s00382-011-1259-y (2013).

6 Rotstayn, L. D. *et al.* Improved simulation of Australian climate and ENSO-related rainfall variability in a global climate model with an interactive aerosol treatment. *International Journal of Climatology* **30**, 1067-1088, doi:10.1002/joc.1952 (2010).

7 Donner, L. J. *et al.* The Dynamical Core, Physical Parameterizations, and Basic Simulation Characteristics of the Atmospheric Component AM3 of the GFDL Global Coupled Model CM3. *Journal of Climate* **24**, 3484-3519, doi:10.1175/2011jcli3955.1 (2011).

8 Schmidt, G. A. *et al.* Present-Day Atmospheric Simulations Using GISS ModelE: Comparison to In Situ, Satellite, and Reanalysis Data. *Journal of Climate* **19**, 153-192, doi:10.1175/jcli3612.1 (2006).

9 Collins, W. J. *et al.* Development and evaluation of an Earth-System model – HadGEM2. *Geosci. Model Dev.* **4**, 1051-1075, doi:10.5194/gmd-4-1051-2011 (2011).

10 Volodin, E. M., Dianskii, N. A. & Gusev, A. V. Simulating present-day climate with the INMCM4.0 coupled model of the atmospheric and oceanic general circulations. *Izvestiya, Atmospheric and Oceanic Physics* **46**, 414-431, doi:10.1134/s000143381004002x (2010).

11 Dufresne, J. L. *et al.* Climate change projections using the IPSL-CM5 Earth System Model: from CMIP3 to CMIP5. *Climate Dynamics* **40**, 2123-2165, doi:10.1007/s00382-012-1636-1 (2013).

12 Watanabe, S. *et al.* MIROC-ESM 2010: model description and basic results of CMIP5-20c3m experiments. *Geosci. Model Dev.* **4**, 845-872, doi:10.5194/gmd-4-845-2011 (2011).

13 Yukimoto, S. *et al.* Meteorological Research Institute-Earth System Model Version 1 (MRI-ESM1) - model description. *Technical Report of the Meteorological Research Institute* (2011).

14 Bentsen, M. *et al.* The Norwegian Earth System Model, NorESM1-M – Part 1: Description and basic evaluation of the physical climate. *Geosci. Model Dev.* **6**, 687-720, doi:10.5194/gmd-6-687-2013 (2013).

15 Lauer, A. *et al.* Benchmarking CMIP5 models with a subset of ESA CCI Phase 2 data using the ESMValTool. *Remote Sensing of Environment*, doi:https://doi.org/10.1016/j.rse.2017.01.007 (2017).

**Tables legends**

**Table S1.** CMIP 5 models information. Source:15

**Table S2.** The spatial mean and standard values used for normalizing the soil moisture datasets.

**Figures legends**

**Figure S1.** Scatter plots showing the mean bias between multi-model CMIP5 and observations of each pair of the different variables: (a) CRU and Willmott temperature bias (TAS), (b) CRU and GPCP precipitation bias (pr; mm), and (c) SMOS and CCI spatially normalized soil moisture (SM).

**Figure S2.** Mean bias of summer air temperature (K) CMIP5 models – CRU datasets (1979-2008; JJA).

**Figure S3.** Mean bias of summer precipitation (mm/day) CMIP5 models – GPCP data (1979-2008; JJA).

**Figure S4.** Difference between mean normalized CMIP5 (1978-2008; JJA) and SMOS-IC (2010-2016; JJA) soil moisture data.

**Figure S5.** Difference between mean normalized CMIP5 (1978-2008; JJA) and CCI (2003-2015; JJA) soil moisture data.

**Figure S6.** Link between bias summer air temperature and SMOS soil moisture. a, map relating the SMOS SM bias and the CRU air temperature bias for the CMIP5 multimodel ensemble. Multimodel results reflect results for the CMIP5 ensemble mean. b, maps relating the SMOS soil moisture bias and the CRU air temperature bias for the 20 CMIP5 models. Note that each colour represents a 10% quantile shift (calculated with respect to the spatial histogram of bias results across the CONUS) in both soil moisture and air temperature.

**Figure S7.** Link between biases in CRU summer air temperature and CCI soil moisture. a, maps relating the CCI soil moisture bias and the CRU air temperature bias for the 20 CMIP5 models. b, map relating the CCI soil moisture bias and the CRU air temperature bias for CMIP5 multimodel ensemble. Note that each colour represents a 10% quantile shift in both soil moisture and air temperature.

**Figure S8.** Link between biases in SMOS soil moisture and GPCP precipitation. a, maps relating the SMOS soil moisture bias and the GPCP precipitation bias for the 20 CMIP5 models. b, map relating the SMOS soil moisture bias and the GPCP precipitation bias for CMIP5 multimodel ensemble. Note that each colour represents a 10% quantile shift in both soil moisture and precipitation.

**Figure S9.** Link between biases in CCI soil moisture and GPCP precipitation. a, maps relating the CCI soil moisture bias and the GPCP precipitation bias for the 20 CMIP5 models. b, map relating the CCI soil moisture bias and the GPCP precipitation bias for CMIP5 multimodel ensemble. Note that each colour represents a 10% quantile shift in both soil moisture and precipitation.

**Figure S10.** Cross-correlations between of the mean biases of the three studied variables over the CGP among the CMIP5 models. Top row: air temperature (Temp) bias vs soil moisture (SM) bias. a, Temp bias (Models-CRU) vs SM bias (Models-SMOS-IC). b, Temp bias (Models-Willmott) vs SM bias (Models-SMOS-IC). c, Temp bias (Models-CRU) vs SM bias (Models-CCI). d, Temp bias (Models-Willmott) vs SM bias (Models-CCI). Middle row: Temp bias vs precipitation (P) bias e, Temp bias (Models-CRU) vs Pr bias (Models- CRU). f, Temp bias (Models-Willmott) vs Pr bias (Models- CRU). g, Temp bias (Models-CRU) vs Pr bias (Models- GPCP). h, Temp bias (Models-Willmott) vs Pr bias (Models- GPCP). Bottom row: Pr bias vs SM bias: i, Pr bias (Models-GPCP) vs SM bias (Models-SMOS-IC). j, Pr bias (Models-CRU) vs SM bias (Models-SMOS-IC). k, Pr bias (Models-GPCP) vs SM bias (Models-CCI). l, Pr bias (Models-CRU) vs SM bias (Models-CCI). The inter-model correlation R and p-value are shown on each panel.

**Figure S11.** Taylor diagram comparing CCI and SMOS soil moisture retrievals to ARM in situ observations using daily values available within the period 2010-2017. Note that the centred RMSE and standard deviation (SD) are normalized by the standard deviation of the observations.
